# Phytochemical profiling and insecticidal activity of *Moricandia sinaica* across growth stages: experimental and molecular docking insights

**DOI:** 10.1002/jsfa.70956

**Published:** 2026-08-03

**Authors:** Roukia Zatout, Ouided Benslama, Chaima Zatout, Noreddine Kacem Chaouche, Stefania Garzoli

**Affiliations:** ^1^ Department of Microbial Biotechnology, Faculty of Natural and Life Science University of Blida 1 Blida Algeria; ^2^ Département de Biologie Appliquée, Laboratoire de Mycologie, de Biotechnologie et de l'Activité Microbienne (LaMyBAM) Université des Frères Mentouri Constantine Algeria; ^3^ Laboratory of Biological Engineering, Valorization, and Innovation of Agri‐Food Products University of Larbi Ben M'hidi Oum El Bouaghi Algeria; ^4^ Research Unit of Environmental and Structural Molecular Chemistry University of Constantine (Mentouri) Constantine Algeria; ^5^ Department of Biology, Faculty of Natural and Life Science University of Constantine (Mentouri) Constantine Algeria; ^6^ Department of Chemistry and Technologies of Drug Sapienza University Rome Italy

**Keywords:** fatty acids, GNBP3, insecticidal activity, molecular docking, *Moricandia sinaica*, *Plodia interpunctella*, volatile compounds

## Abstract

**BACKGROUND:**

Stored food products are highly vulnerable to infestation by insects and mites, resulting in significant postharvest losses worldwide. Plant‐derived bioactive compounds represent promising eco‐friendly alternatives to synthetic pesticides. This study investigated the phytochemical composition of *Moricandia sinaica* at different growth stages and evaluated its insecticidal activity against *Plodia interpunctella* larvae, complemented by molecular docking analysis.

**RESULTS:**

Gas chromatography–mass spectrometry analysis revealed clear stage‐dependent variations in volatile composition. At the flowering stage, hexahydrofarnesylacetone, 1,8‐cineole, and *α*‐thujene were predominant, whereas mature plants were characterized mainly by hexahydrofarnesylacetone, *α*‐pinene, and dihydroactinidiolide. Fatty acid analysis showed that palmitic acid remained the dominant component, although it decreased, while oleic and stearic acids increased at maturity. Insecticidal bioassays showed concentration‐ and time‐related increases in larval mortality against *P. interpunctella* larvae. Molecular docking suggested that major fatty acids may interact with residues located within the carbohydrate‐recognition region of *β*‐1,3‐glucan‐binding protein 3 (GNBP3), providing a predictive hypothesis that requires further experimental validation.

**CONCLUSION:**

The results highlight *Moricandia sinaica* as a promising source of bioactive compounds with potential applications in environmentally friendly pest management strategies for stored products. © 2026 The Author(s). *Journal of the Science of Food and Agriculture* published by John Wiley & Sons Ltd on behalf of Society of Chemical Industry.

## INTRODUCTION

Stored food products are highly susceptible to pests such as storage insects and mites, which cause significant postharvest losses worldwide.^1^, ^2^ Identifying natural plant‐derived compounds that can protect these products is therefore of great practical importance.

Plants produce a diverse array of metabolites that contribute to their development and ecological interactions.^3^ In particular, secondary metabolites such as phenolics, terpenoids, and volatile compounds play key roles in defense mechanisms and environmental adaptation. The composition and abundance of these compounds vary depending on species, environmental conditions, and developmental stage, thereby influencing the biological activity of plant extracts.^4^, ^5^, ^6^

In Algeria, large regions are covered by semiarid and arid ecosystems.^7^, ^8^ Plants in these areas face harsh conditions such as prolonged drought, extreme temperature fluctuations, and nutrient‐poor soils. Such stresses often stimulate the accumulation of protective metabolites, making these plants promising sources of bioactive compounds with potential applications in pest management and food preservation.^9^, ^10^, ^11^

*Moricandia sinaica* is a wild Brassicaceae species naturally adapted to semiarid habitats, yet it remains largely unexplored from a chemical and biological perspective. In Algeria, this species plays an ecological role in adapting to extreme arid conditions, which may contribute to the production of stress‐related bioactive metabolites.

Plants within the Brassicaceae family are well known for their rich arsenal of defensive metabolites, particularly glucosinolates and their breakdown products, such as isothiocyanates, nitriles, and thiocyanates.^12^ These compounds play central roles in plant tolerance to drought, heat, and herbivore pressure. Several members of the family – such as *Brassica nigra*, *Armoracia rusticana* (horseradish), *Sinapis alba* (white mustard), and *Eruca sativa* (rocket) – have demonstrated strong insecticidal or repellent activities through their volatile isothiocyanates.^13^, ^14^, ^15^, ^16^ These volatiles can disrupt insect feeding, inhibit larval development, or reduce infestation in stored grains. Given these well‐documented defense strategies in related species, profiling the metabolite composition of *M. sinaica* throughout its growth stages may reveal unique or enhanced defensive compounds with promising potential for protecting stored food products from postharvest pests. Despite its adaptation to semiarid ecosystems, *M. sinaica* remains poorly explored compared to other Brassicaceae species, highlighting its potential as a novel source of bioactive compounds.

Recent advances in computational tools, such as molecular docking, have provided valuable insights into the interactions between plant‐derived molecules and biological targets. Integrating *in silico* predictions with laboratory bioassays enables a more complete evaluation of the insecticidal potential of plant extracts. The *β*‐1,3‐glucan‐binding protein 3 (GNBP3) was selected as a molecular target because of its reported involvement in insect innate immunity, particularly in *β*‐1,3‐glucan recognition and activation of the prophenoloxidase defense pathway. In this context, docking analysis was used only as a predictive tool to explore possible interactions between selected phytochemicals and the GNBP3 binding region.

The study reported here aimed to provide a comprehensive analysis of *M. sinaica* by combining molecular identification, chemical profiling, insecticidal bioassays, and molecular docking. First, molecular identification was performed to confirm the species identity. Then, volatile compounds and fatty acids were analyzed at the flowering and mature stages to evaluate developmental variation in chemical composition. The insecticidal activity of *n*‐hexane and dichloromethane extracts was assessed against third‐instar larvae of *Plodia interpunctella* using fumigant and residual toxicity assays. Finally, molecular docking was performed as a predictive approach to explore possible interactions between selected phytochemicals and GNBP3. We hypothesized that the developmental stage of *M. sinaica* influences its phytochemical composition and may affect its insecticidal potential.

## MATERIALS AND METHODS

### Plant material

Samples of *M. sinaica* were collected from semiarid regions of Ghardaia, Algeria (32.4906° N, 3.6736° E; altitude: 566 m), during different growth stages in April 2025 (flowering and mature stages). The flowering stage was defined as the period when approximately 50% of the flowers were fully open, whereas the mature stage corresponded to plants bearing fully developed and dry seed pods.

Based on regional meteorological data for Ghardaia, the average temperature during April 2025 ranged between 25 and 30 °C, while the monthly rainfall remained below 10 mm, reflecting the typical semiarid climatic conditions of the region. The plant material was authenticated based on morphological characteristics (leaf shape, stem structure, flowers, and seed pods) and further confirmed by molecular identification using DNA barcoding of the ITS region.^17^, ^18^, ^19^

To ensure biological variability, samples were collected from at least three independent plants growing in different locations within the study area and processed separately.

Fresh aerial parts were carefully washed with distilled water, air‐dried under ambient climatic conditions for 15 days, and then ground into a fine powder using a laboratory mill. The powdered material was stored in airtight containers at 4 °C until further extraction.

### Preparation of plant extracts

*n*‐Hexane and dichloromethane (DCM) extracts were obtained using a simple maceration method. Briefly, 50 g of the powdered plant material was soaked in 500 mL of the respective solvent (*n*‐hexane or DCM) at room temperature for 72 h, with occasional gentle shaking to enhance extraction. The resulting extracts were filtered through Whatman No. 1 filter paper and concentrated under reduced pressure using a rotary evaporator set at 40 °C.^20^ The dried residues were weighed, transferred into amber vials, and stored at 4 °C until further chemical analysis and biological testing.^21^

### HS‐SPME‐GC–MS analysis

The volatile composition of *M. sinaica* dry powders was analyzed using HS‐SPME‐GC–MS. Briefly, 0.5 g of sample was placed in a sealed vial and equilibrated at 60 °C. Volatiles were extracted using a DVB/CAR/PDMS fiber and analyzed using a gas chromatography–mass spectrometry (GC–MS) system (Clarus 500, PerkinElmer) equipped with a VF‐5 capillary column. The oven program ranged from 45 to 220 °C at 6° min^−1^ and finally held for 15 min. Compounds were identified by comparison of mass spectra with NIST and Wiley libraries and by linear retention indices (C_8_–C_25_ *n*‐alkanes). Relative abundances were expressed as percentage peak areas.

### Fatty acid analysis

Fatty acid composition was determined using GC–MS after lipid extraction and derivatization to fatty acid methyl esters (FAMEs). Briefly, lipids were extracted using chloroform/methanol (2:1, v/v) and transmethylated with BF_3_–methanol. FAMEs were analyzed using GC–MS (Clarus 500, PerkinElmer). The GC conditions were as follows: the injector was set at 280 °C and the oven temperature program started from 170 °C and increased up to 260 °C with a rate of 3 °C min^−1^ and held constant for 15 min. Fatty acids were identified by comparison with spectral libraries and retention indices and the relative abundances were expressed as percentage peak areas. Analyses were performed in triplicate.

### Insecticidal assay

The insecticidal potential of *M. sinaica* was evaluated at the mature stage, when the plant is known to contain the highest levels of defensive metabolites. *n*‐Hexane and DCM extracts were tested against *Plodia interpunctella* (Indian meal moth), a major pest of stored grains and dried foods, responsible for significant postharvest losses. Dried extracts obtained after solvent evaporation were re‐dissolved in their respective solvents, *n*‐hexane or DCM, to prepare stock solutions at 100 mg mL^−1^. From these stock solutions, serial dilutions were prepared to obtain test solutions at 25, 50, 75, and 100 mg mL^−1^. These concentrations were used for both fumigant and residual toxicity assays.

#### Insect rearing

Colonies of *P. interpunctella* were maintained under controlled laboratory conditions and fed a diet of wheat flour supplemented with 10 g of moist yeast per kilogram of flour. Larvae were reared at 27 ± 2 °C and 80 ± 5% relative humidity. Only third‐instar larvae were used in bioassays to ensure a uniform developmental stage.^22^

#### Fumigant toxicity assay

Fumigant toxicity was assessed in 9 cm Petri dishes used as sealed exposure chambers. Whatman No. 1 filter paper discs were affixed to the lids and treated with 100 μL of extract solution at concentrations of 25, 50, 75, and 100 mg mL^−1^. Based on the applied volume, these treatments corresponded to 2.5, 5.0, 7.5, and 10.0 mg of extract per chamber, respectively. The dishes were left open for 5–10 min to allow solvent evaporation. Ten third‐instar larvae were introduced into each chamber, which was then tightly sealed. Controls included solvent‐only and untreated dishes. Each treatment was conducted in triplicate.^23^ This dosing approach allowed the fumigant exposure to be expressed as the amount of extract applied per chamber.

Mortality was recorded at 24, 48, and 72 h and expressed as the raw number of dead larvae per replicate. The results are presented as mean numbers of dead larvae ± standard deviation (SD) from three independent replicates, with ten larvae used per replicate.

#### Residual toxicity assay

For contact toxicity, 1 mL of each extract was applied to filter paper lining Petri dish bases. After 10 min of solvent evaporation, ten larvae were introduced. Negative controls included solvent‐treated and distilled water‐treated papers. Each treatment was conducted in triplicate.

The applied concentrations corresponded to 25, 50, 75, and 100 mg mL^−1^, ensuring reproducible and quantifiable exposure to the dried extract.

Mortality was recorded at 24, 48, and 72 h and expressed as the raw number of dead larvae per replicate. The results are presented as mean numbers of dead larvae ± SD from three independent replicates, with ten larvae used per replicate.

### Statistical analysis

Data are presented as mean ± SD of three independent replicates. Mortality values correspond to the raw number of dead larvae per replicate, with ten larvae used in each replicate. Decimal values result from averaging replicate counts and do not represent fractional individuals. The results were reported as raw mortality counts rather than corrected mortality percentages.

Statistical analysis was performed separately for each toxicity assay, namely fumigant toxicity and residual toxicity, and for each exposure time, namely 24, 48, and 72 h. Within each assay and exposure time, one‐way analysis of variance (ANOVA) followed by Tukey's HSD test was used to compare treatments. This approach was used to identify differences among treatments at each observation time rather than to model the complete factorial effects of extract type, concentration, exposure time, or their interactions. Differences were considered statistically significant at *P* < 0.05.

LT₅₀ values were not calculated because the limited number of observation time points, namely 24, 48, and 72 h, did not allow reliable estimation through probit analysis.

### Molecular docking study

#### Protein preparation

The three‐dimensional structure of GNBP3 from *P. interpunctella* (Protein Data Bank identifier: 3AQZ) was retrieved from the Protein Data Bank. The protein was prepared using UCSF Chimera v1.16, where water molecules, redundant chains, and nonessential heteroatoms were removed. Hydrogen atoms were added according to the AMBER ff14SB force field, and charges were assigned using the Gasteiger method.^24^ The co‐crystallized ligand (laminarihexaose) was retained for re‐docking validation.

#### Ligand preparation

Major phytochemical constituents of *M. sinaica* identified through chemical analysis were selected for docking. Their three‐dimensional structures were obtained from the PubChem database in SDF format, energy‐minimized, and converted to PDBQT format using UCSF Chimera v1.16.^25^

#### Docking protocol

Molecular docking was performed using AutoDock Vina integrated into Chimera v1.16.^24^ The binding site was defined based on the coordinates of the co‐crystallized laminarihexaose. Grid box dimensions and center were optimized to fully encompass the carbohydrate‐recognition domain.

#### Validation by re‐docking

To validate the docking protocol, the co‐crystallized ligand was removed and re‐docked into the same binding pocket. The root mean square deviation between the experimental and re‐docked pose was 1.812 Å, confirming the reliability and reproducibility of the docking setup.^26^, ^27^

#### Visualization and interaction analysis

Docking poses and protein–ligand interactions were analyzed and visualized using Discovery Studio Visualizer v21.1.^28^, ^29^ Hydrogen bonds, hydrophobic interactions, electrostatic contacts, and amino acid residue involvement were systematically annotated for comparison with the natural ligand.

## RESULTS AND DISCUSSION

### Morphological and molecular identification of *M. sinaica*

Morphological examination confirmed that the collected specimens exhibited the characteristic diagnostic features of *M. sinaica*, including its distinct leaf morphology, stem structure, and floral traits. These findings are consistent with previous descriptions of the species within the Brassicaceae family.^30^, ^31^, ^32^ Molecular analysis further supported these observations, where ITS region barcoding unequivocally validated the species identity, in agreement with earlier studies demonstrating the effectiveness of ITS markers for species discrimination in *Moricandia* and related genera.^11^, ^33^ The verified ITS sequence has been deposited in GenBank under the accession number PX627879, providing a reliable genetic reference for future biochemical and taxonomic studies.

### Volatile chemical composition of *M. sinaica*

The volatile profile of *M. sinaica* was analyzed at two developmental stages: flowering and mature. At the flowering stage, nine volatile compounds were identified (Table 1). Hexahydrofarnesylacetone was predominant (67.1%), followed by 1,8‐cineole (10.7%) and *α*‐thujene (10.4%). Minor components included limonene (5.5%), *α*‐pinene (1.8%), 1,2,4,5‐tetramethylbenzene (1.6%), propanamide (1.2%), pentanal (1.0%), and dihydroactinidiolide (0.7%). The high concentration of hexahydrofarnesylacetone suggests a significant role in the plant's biological activities, particularly its insecticidal properties.

**Table 1:** Chemical volatile composition of *Moricandia sinaica* at the flowering stage (FS) and mature stage (MS). Relative abundances are expressed as percentage peak areas (mean ± SD)

No.	Component	LRI^calc^	LRI^lit^	FS (%)	MS (%)
1	Pentanal	689	692	1.0 ± 0.08	1.1 ± 0.25
2	Propanamide	918	921	1.2 ± 0.13	1.3 ± 0.14
3	*α*‐Thujene	926	923	10.4 ± 1.05	—
4	*α*‐Pinene	938	941	1.8 ± 0.70	20.1 ± 3.05
5	1,2,4,5‐Tetramethylbenzene	1096	1099	1.6 ± 0.62	—
6	Limonene	1019	1023	5.5 ± 0.24	—
7	1,8‐Cineole	1035	1032	10.7 ± 1.11	3.3 ± 0.41
8	Dihydroactinidiolide	1526	1520	0.7 ± 0.07	11.1 ± 1.32
9	Hexahydrofarnesylacetone	1844	1846	67.1 ± 6.22	63.1 ± 5.36
	*Total*			100.0	100.0

Components are listed according to their elution order on the apolar column.^2^ LRI^calc^, linear retention indices measured on apolar column^3^; LRI^lit^, linear retention indices from the literature. Relative abundances correspond to GC–MS peak area percentages.

At the mature stage, the volatile composition shifted slightly (Table 1). Hexahydrofarnesylacetone remained dominant (63.1%), while *α*‐pinene (20.1%) and dihydroactinidiolide (11.1%) increased. Other detected compounds included 1,8‐cineole (3.3%), propanamide (1.3%), and pentanal (1.1%). Notably, *α*‐thujene and limonene were absent at this stage. These changes indicate that volatile composition is developmentally regulated in *M. sinaica*.

Hexahydrofarnesylacetone, present at both stages, is a bioactive sesquiterpenoid reported in other Brassicaceae species with insecticidal and antimicrobial effects.^34^, ^35^ Its persistence suggests a key role in plant defense and potential as a chemical marker for *M. sinaica*. *α*‐Thujene, detected only at flowering, is a monoterpene with insecticidal and antifungal activity, capable of deterring stored‐product pests and some phytopathogenic fungi.^36^, ^37^, ^38^, ^39^ Its absence in mature plants may reflect developmental regulation or volatilization. *α*‐Pinene increased substantially from 1.8% at flowering to 20.1% at maturity. Known for insecticidal, antimicrobial, and antioxidant properties,^40^, ^41^, ^42^ its higher concentration at maturity may enhance defense against pests such as *Stegobium paniceum*. 1,8‐Cineole decreased from 10.7% to 3.3% between flowering and maturity; this monoterpene exhibits antimicrobial and fumigant activity.^43^, ^44^ The reduction may indicate a shift in defense strategy toward compounds that accumulate later, such as *α*‐pinene and dihydroactinidiolide. Dihydroactinidiolide increased markedly from 0.7% at flowering to 11.1% at maturity. Derived from carotenoids, it is recognized for insect‐repellent and antifungal properties,^35^, ^45^ which may contribute to the higher insecticidal activity observed in mature plant extracts.

The differences in volatile composition between flowering and mature stages indicate developmental regulation of secondary metabolites, with certain compounds increasing at maturity to enhance insecticidal and antimicrobial defenses. These findings are consistent with previous reports in Brassicaceae and other aromatic plants, where monoterpenes and sesquiterpenes generally accumulate during maturation to strengthen defense against herbivores and pathogens.^46^, ^47^

### Fatty acid profile of *M. sinaica*

Fatty acid composition was analyzed at flowering and mature stages (Table 2). As evident from Table 2, palmitic acid was the dominant component at both stages, while oleic and stearic acids increased at the mature stage. At the flowering stage, palmitic acid was the predominant fatty acid, while oleic and stearic acids were present at lower levels. At the mature stage, a decrease in palmitic acid was observed, accompanied by an increase in oleic and stearic acids. Myristic acid appeared at 1.9%. These changes suggest a developmental shift in lipid metabolism, possibly reflecting adaptation to environmental stress and preparation for reproductive or defensive functions.

**Table 2:** Fatty acid composition of *Moricandia sinaica* at the flowering stage (FS) and mature stage (MS)

No.	Fatty acids	LRI^calc^	LRI^lit^	FS (%)	MS (%)
1	Myristic acid, C14:0	1755	1752	—	1.9 ± 0.11
2	Palmitic acid, C16:0	1968	1973	72.7 ± 6.95	59.4 ± 4.96
3	Oleic acid, C18:1*n*9	2173	2171	17.0 ± 2.01	23.2 ± 3.10
4	Stearic acid, C18:0	2181	2178	10.2 ± 1.05	15.4 ± 1.22
	Total identified fatty acids			99.9	99.9
	Saturated fatty acids			82.9	76.7
	Unsaturated fatty acids			17.0	23.2

Relative abundances are expressed as percentage peak areas (mean ± SD). The components are reported according to their elution order on apolar column (VF‐5ms); LRI^calc^, linear retention indices measured on apolar column; LRI^lit^, linear retention indices from the literature. Relative abundances correspond to GC–MS peak area percentages.

These changes indicate a developmental shift in fatty acid composition, possibly reflecting modifications in lipid metabolism during maturation, as previously reported in Brassicaceae and other plant species.^48^, ^49^ The increase in oleic and stearic acids at the mature stage may contribute to the biological properties of the extract, since fatty acids have been reported to possess bioactive potential.^48^ However, this relationship should be interpreted cautiously because compound‐level bioassays were not performed. Meanwhile, the persistent predominance of palmitic acid suggests that this fatty acid remains a major component of the lipid fraction of *M. sinaica* throughout development.

### Insecticidal assays

#### Fumigant toxicity assay

Both *n*‐hexane and DCM extracts induced mortality in *P. interpunctella* larvae in a concentration‐ and time‐related manner (Table 3). Mortality values are expressed as the mean number of dead larvae (± SD) per replicate (10 larvae per replicate). Control mortality remained low (0.67 ± 0.58 dead larvae).

**Table 3:** Fumigant toxicity of *n*‐hexane and DCM extracts of *Moricandia sinaica* against third‐instar larvae of *Plodia interpunctella*, expressed as the mean number of dead larvae per replicate

Source	Extract amount (mg per chamber)	Number of dead larvae (mean ± SD)		
		24 h	48 h	72 h
Control	—	0.67 ± 0.58 ^c^	0.67 ± 0.58 ^c^	0.67 ± 0.58 ^c^
*n*‐Hexane	2.5	2.33 ± 0.58 ^bc^	3.00 ± 0.00 ^bc^	4.00 ± 0.00 ^b^
*n*‐Hexane	5.0	3.67 ± 0.58 ^b^	5.00 ± 0.58 ^b^	6.00 ± 0.00 ^ab^
*n*‐Hexane	7.5	5.00 ± 0.00 ^ab^	6.00 ± 0.58 ^a^	7.00 ± 0.00 ^a^
*n*‐Hexane	10.0	6.00 ± 0.00 ^a^	6.67 ± 0.58 ^a^	8.00 ± 0.00 ^a^
DCM	2.5	2.67 ± 0.58 ^bc^	3.67 ± 0.58 ^b^	4.00 ± 1.00 ^b^
DCM	5.0	3.67 ± 0.58 ^ab^	4.33 ± 0.58 ^a^	5.67 ± 0.58 ^a^
DCM	7.5	5.67 ± 0.58 ^a^	6.67 ± 0.58 ^a^	7.67 ± 0.58 ^a^
DCM	10.0	7.00 ± 0.58 ^a^	8.33 ± 0.58 ^a^	8.67 ± 0.58 ^a^

Values represent the mean number of dead larvae ± SD from three independent replicates, with ten larvae used per replicate. Decimal values arise from averaging replicate counts and do not represent fractional individuals. Different letters indicate significant differences among treatments within the same exposure time according to one‐way ANOVA followed by Tukey's HSD test (*P* < 0.05). In the fumigant assay, extract amount was expressed as milligrams per chamber because 100 μL of each extract solution was applied to the filter paper disc fixed to the Petri dish lid.

For the *n*‐hexane extract, mortality increased from 2.33 ± 0.58 dead larvae at 2.5 mg per chamber to 6.00 ± 0.00 dead larvae at 10.0 mg per chamber after 24 h, reaching 8.00 ± 0.00 dead larvae at 72 h. Similarly, the DCM extract caused increasing mortality with extract amount, ranging from 2.67 ± 0.58 dead larvae at 2.5 mg per chamber to 7.00 ± 0.58 dead larvae at 10.0 mg per chamber after 24 h, reaching 8.67 ± 0.58 dead larvae at 72 h.

#### Residual toxicity assay

Both extracts exhibited contact toxicity, with mortality increasing with both concentration and exposure duration (Table 4). Mortality values represent the mean number of dead larvae (± SD) per replicate (10 larvae per replicate).

**Table 4:** Residual toxicity of *n*‐hexane and DCM extracts of *Moricandia sinaica* against third‐instar larvae of *Plodia interpunctella*, expressed as the mean number of dead larvae per replicate

Source	Extract concentration (mg mL^−1^)	Number of dead larvae (mean ± SD)		
		24 h	48 h	72 h
Control	—	0.33 ± 0.58 ^c^	0.67 ± 0.58 ^c^	0.67 ± 0.58 ^c^
*n*‐Hexane	25	2.00 ± 0.58 ^b^	2.67 ± 0.58 ^b^	3.67 ± 0.58 ^b^
*n*‐Hexane	50	3.33 ± 0.58 ^ab^	4.67 ± 0.58 ^ab^	5.67 ± 0.58 ^ab^
*n*‐Hexane	75	4.33 ± 0.00 ^a^	5.33 ± 0.58 ^a^	6.33 ± 0.58 ^a^
*n*‐Hexane	100	6.00 ± 0.00 ^a^	6.67 ± 0.58 ^a^	8.00 ± 0.00 ^a^
DCM	25	2.33 ± 0.58 ^b^	3.33 ± 0.58 ^b^	3.67 ± 0.58 ^b^
DCM	50	3.33 ± 0.58 ^ab^	4.33 ± 0.58 ^ab^	5.67 ± 0.58 ^a^
DCM	75	5.33 ± 0.58 ^a^	6.33 ± 0.58 ^a^	7.33 ± 0.58 ^a^
DCM	100	6.33 ± 0.58 ^a^	7.67 ± 0.58 ^a^	7.67 ± 0.58 ^a^

Values represent the mean number of dead larvae ± SD from three independent replicates, with ten larvae used per replicate. Decimal values arise from averaging replicate counts and do not represent fractional individuals. Different letters indicate significant differences among treatments within the same exposure time according to one‐way ANOVA followed by Tukey's HSD test (*P* < 0.05). In the residual toxicity assay, treatments were expressed as extract concentration (mg mL^−1^) because 1 mL of each extract solution was applied to the filter paper lining the Petri dish base.

The *n*‐hexane extract induced moderate mortality at the lowest tested concentration, 25 mg mL^−1^, with 2.00 ± 0.58 dead larvae after 24 h, progressively rising to 8.00 ± 0.00 dead larvae at 100 mg mL^−1^ after 72 h. Similarly, the DCM extract demonstrated residual effects, reaching 7.67 ± 0.58 dead larvae at 100 mg mL^−1^ after 72 h, indicating contact toxicity against *P. interpunctella* larvae.

Both *n*‐hexane and DCM extracts of *M. sinaica* showed larvicidal activity against third‐instar larvae of *P. interpunctella*, with mortality generally increasing with concentration and exposure time. The chemical data obtained from the plant material indicated the presence of fatty acids such as palmitic, oleic, stearic, and myristic acids, as well as volatile constituents including hexahydrofarnesylacetone, *α*‐pinene, dihydroactinidiolide, 1,8‐cineole, pentanal, and propanamide. These compounds have previously been associated with insecticidal or bioactive properties and may contribute to the activity observed in the present study.^50^, ^51^, ^52^, ^53^

However, the relationship between the detected chemical constituents and the mortality recorded in the bioassays should be interpreted with caution. The chemical profiles reported here describe the plant material at different growth stages and do not provide compound‐by‐compound quantification for each tested extract. Therefore, the contribution of individual compounds or compound classes to fumigant activity or residual toxicity remains inferential. The observed activity may result from the combined effects of several constituents, possible synergistic interactions, and solvent‐dependent extraction of lipophilic or volatile compounds.^54^, ^55^, ^56^

Accordingly, while the higher fumigant activity of the DCM extract and the residual activity of the *n*‐hexane extract are consistent with the presence of volatile and lipophilic constituents, respectively, these associations cannot be considered direct evidence of causality. Further extract‐specific chemical profiling, fractionation, and bioassays using isolated compounds are required to confirm the active constituents and clarify their respective contributions to the insecticidal activity of *M. sinaica*. Therefore, the chemical–bioactivity relationship proposed in this study should be considered exploratory rather than confirmatory.

### Molecular docking

GNBP3 of *P. interpunctella* was selected as the molecular target for this docking analysis because of its reported involvement in insect innate immunity.^57^ GNBP3 is a pattern‐recognition receptor involved in *β*‐1,3‐glucan recognition and activation of immune‐related responses in insects.^58^, ^59^ Because of this reported biological role, it was selected as a molecular target to explore possible interactions with selected phytochemicals of *M. sinaica*. However, the docking analysis was used only as a predictive approach and does not demonstrate immune pathway disruption or a confirmed mechanism of insecticidal activity.

In this study, selected major phytochemical constituents identified in the plant material were submitted to molecular docking. The docking results of the co‐crystallized ligand, laminarihexaose, and the selected compounds, including the major volatile compound hexahydrofarnesylacetone and the predominant fatty acids palmitic, oleic, and stearic acids, are summarized in Table 5 and visually represented in Fig. 1.

**Table 5:** Binding energies and interaction profiles of the co‐crystallized ligand (laminarihexaose) and the best‐scoring phytochemical constituents of *Moricandia sinaica* docked into the carbohydrate‐recognition domain of GNBP3 of *Plodia interpunctella* (Protein Data Bank identifier: 3AQZ)

			Binding energy (kcal mol^−1^)	Hydrogen interactions (distance in Å)	Hydrophobic interactions	Electrostatic interactions
GNBP3 (3AQZ)	Co‐crystallized ligand	Laminarihexaoses	−6.7	HIS31 (2.77), ARG87 (2.57, 2.65, 3.10), CYS11 (3.06), CYS8 (2.67, 1.95), TYR78 (2.56), GLY85 (2.39), HIS31 (2.88), ARG48 (2.84), CYS12 (3.29), ALA47 (2.37, 3.46, 2.58), LEU41 (2.89, 2.46), TYR78 (2.46), CYS6 (2.67, 2.40)	—	—
	Best docked compounds	Palmitic acid	−5.7	ARG87 (2.74), GLY85 (2.87)	ALA29 (alkyl), LEU27 (alkyl), HIS31 (*π*–alkyl), TRP76 (*π*–alkyl), TYR78 (*π*–alkyl)	ARG87 (salt bridge; attractive charge)
		Oleic acid	−5.4	ARG87 (2.08, 2.23)	LEU41 (alkyl), HIS31 (*π*–alkyl), TRP76 (*π*–alkyl), TYR78 (*π*–alkyl)	ARG87 (salt bridge, attractive charge)
		Stearic acid	−5.2	ARG87 (2.05, 2.57)	ALA47 (alkyl), HIS31 (*π*–alkyl), TYR78 (*π*–alkyl)	ARG87 (attractive charge)
		Hexahydrofarnesylacetone	−4.9	—	ALA29 (alkyl), ALA47 (alkyl), LEU27 (alkyl), HIS31 (*π*–alkyl), TYR78 (*π*–alkyl)	—

**Figure 1:**
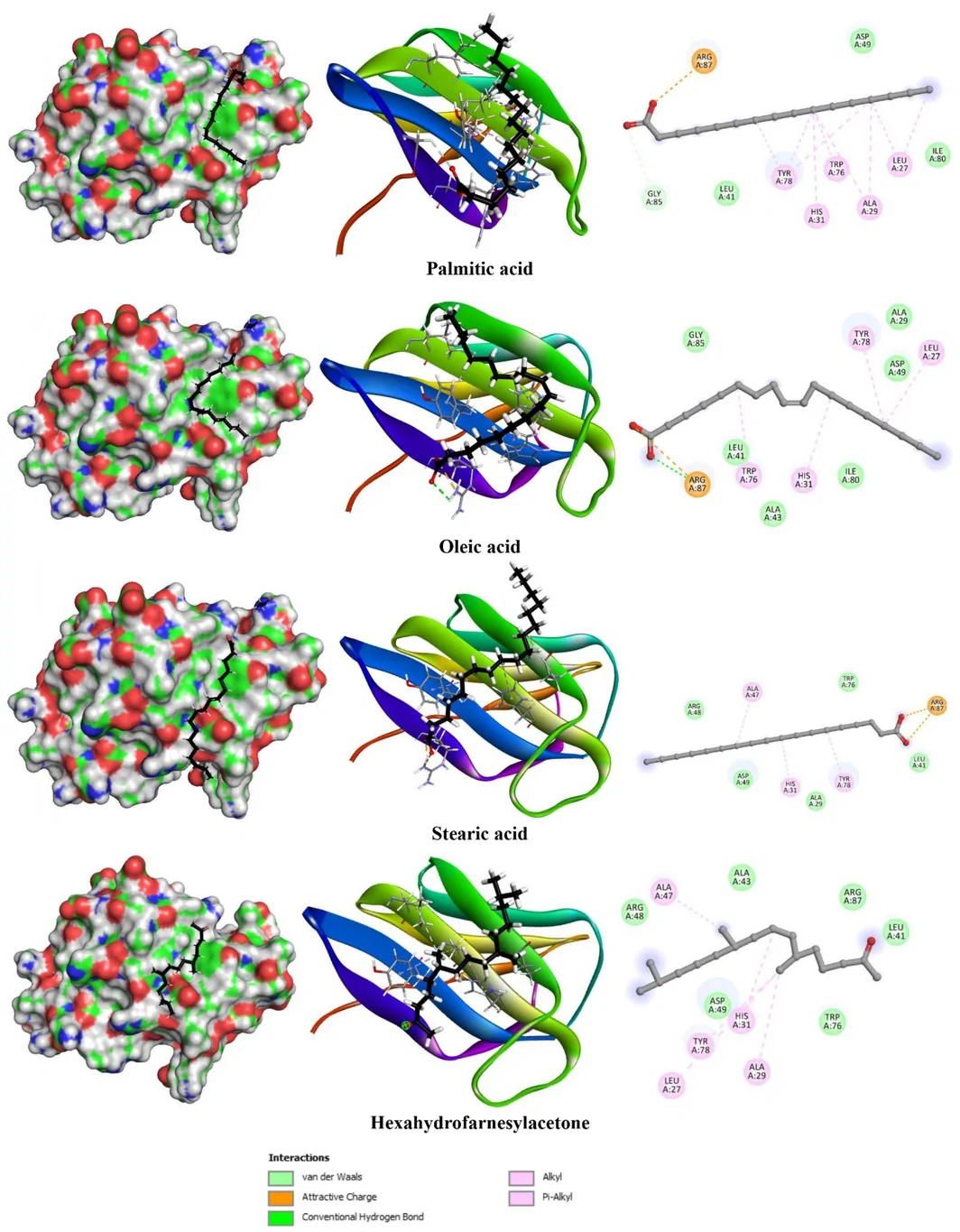
Predicted binding modes of the selected phytochemical compounds, including hexahydrofarnesylacetone and the major fatty acids (palmitic, oleic, and stearic acids), within the carbohydrate‐recognition domain of GNBP3 of *Plodia interpunctella* (Protein Data Bank identifier: 3AQZ). For each ligand, the left‐hand panel shows the molecular surface representation of GNBP3 highlighting the binding pocket; the middle panel illustrates the docked pose within the protein's secondary structure; and the right‐hand panel displays the two‐dimensional interaction diagram summarizing key hydrogen bonds, hydrophobic contacts, and electrostatic interactions with the surrounding amino acid residues.

Laminarihexaose was used as a reference ligand to define the key residues of the natural binding pocket and to provide a structural benchmark for comparing the binding behavior of the tested phytochemicals.

Analysis of the interaction patterns showed that the selected fatty acids were predicted to bind within the carbohydrate‐recognition region of GNBP3 and to interact with residues also involved in the binding of the co‐crystallized ligand. Among these residues, ARG87 appeared frequently in the predicted interaction profiles of palmitic, oleic, and stearic acids. This residue was involved in hydrogen‐bond or electrostatic interactions with the fatty acids, suggesting that it may contribute to ligand stabilization within the modeled binding pocket.

The binding energies followed the order palmitic acid (−5.7 kcal mol^−1^), oleic acid (−5.4 kcal mol^−1^), and stearic acid (−5.2 kcal mol^−1^), indicating moderate predicted affinity for the GNBP3 binding region. Palmitic acid showed the most favorable docking score among the tested phytochemicals and formed predicted hydrogen‐bond interactions with ARG87 and GLY85, together with hydrophobic contacts involving ALA29, LEU27, HIS31, TRP76, and TYR78. Oleic acid also interacted with ARG87 and formed hydrophobic interactions with LEU41, HIS31, TRP76, and TYR78. Stearic acid showed predicted interactions with ARG87 and hydrophobic contacts involving ALA47, HIS31, and TYR78. These interaction patterns suggest that the fatty acids may occupy regions close to the carbohydrate‐recognition site, although this does not demonstrate functional inhibition of GNBP3.

Hexahydrofarnesylacetone, the major volatile compound detected in *M. sinaica*, showed a lower predicted binding affinity (−4.9 kcal mol^−1^) and interacted mainly through hydrophobic contacts with residues such as ALA29, ALA47, LEU27, HIS31, and TYR78. The absence of predicted hydrogen bonds or electrostatic interactions indicates a less specific interaction pattern compared with the fatty acids.

Overall, the docking results suggest that selected fatty acids from *M. sinaica* may interact with residues located within or near the carbohydrate‐recognition region of GNBP3. These findings provide a possible structural hypothesis that may help guide future studies on the interaction between plant‐derived fatty acids and insect immune‐related proteins. However, these *in silico* results do not confirm competitive inhibition, immune pathway disruption, or a direct GNBP3‐mediated mechanism of insecticidal activity. Therefore, the docking data should be interpreted strictly as predictive and hypothesis‐generating. Additional biochemical assays, binding experiments, gene expression analyses, or *in vivo* validation are required to determine whether these predicted interactions have functional relevance in *P. interpunctella* larvae.

## CONCLUSIONS

This study showed that *Moricandia sinaica* exhibits stage‐dependent variations in its chemical composition, which may be associated with its observed insecticidal activity against *Plodia interpunctella*. The volatile profile was dominated by hexahydrofarnesylacetone (63.1–67.1%) at both stages, while *α*‐pinene showed a marked increase at maturity, reaching 20.1%. In parallel, fatty acid analysis revealed a decrease in palmitic acid (from 72.7% to 59.4%) and an increase in oleic acid (17.0–23.2%) and stearic acid (10.2–15.4%) at the mature stage. Biological assays confirmed a concentration‐ and time‐dependent insecticidal effect, with mortality reaching up to 8.67 dead larvae out of 10 after 72 h. The DCM extract showed higher fumigant toxicity, whereas the *n*‐hexane extract displayed progressive residual toxicity. These effects may be associated with the presence of volatile and lipophilic constituents detected in the plant material, but this relationship remains inferential and requires extract‐specific chemical profiling and compound‐level bioassays for confirmation.

Molecular docking analysis predicted that fatty acids, particularly palmitic acid (−5.7 kcal mol^−1^), oleic acid (−5.4 kcal mol^−1^), and stearic acid (−5.2 kcal mol^−1^), may interact with residues located within or near the GNBP3 carbohydrate‐recognition region, especially ARG87. The major volatile compound hexahydrofarnesylacetone displayed mainly hydrophobic contacts within the modeled binding pocket, with a lower predicted affinity (−4.9 kcal mol^−1^). These findings should be interpreted strictly as predictive and hypothesis‐generating, and they do not demonstrate GNBP3 inhibition or immune pathway disruption. Experimental validation through biochemical or *in vivo* assays is required to determine the functional relevance of these predicted interactions.

From an applied perspective, the results suggest that *M. sinaica* extracts could be developed as botanical insecticides, particularly in the form of crude extracts or volatile‐based formulations for fumigation in stored‐product protection systems.

Future studies should focus on isolating individual active compounds, evaluating their specific toxicity, and conducting field‐scale trials to validate their effectiveness under real storage conditions.

## FUNDING INFORMATION

This research received no specific grant from any funding agency in the public, commercial, or not‐for‐profit sectors.

## CONFLICT OF INTEREST

The authors declare no conflict of interest.

## AUTHOR CONTRIBUTIONS

**Roukia Zatout:** Conceptualization, resources, methodology, investigation, writing – original draft, supervision, review and editing; **Ouided Benslama:** Molecular docking, data analysis, writing – original draft; **Noreddine Kacem Chaouche:** Writing – review and editing; **Chaima Zatout:** Practical work, sample preparation, laboratory analysis; **Stefania Garzoli:** Chemical analysis and interpretation, supervision, writing – review and editing.

## Data Availability

The data that support the findings of this study are available from the corresponding author upon reasonable request.
